# Case report: Synchronous bladder and retroperitoneal paragangliomas: an extremely rare entity

**DOI:** 10.3389/fonc.2025.1593934

**Published:** 2025-08-13

**Authors:** Weitao Huang, Yiming Liu, Miaoping Zhou

**Affiliations:** ^1^ Department of Radiology, The Quzhou Affiliated Hospital of Wenzhou Medical University, Quzhou People’s Hospital, Quzhou, China; ^2^ Department of Pathology, The Quzhou Affiliated Hospital of Wenzhou Medical University, Quzhou People’s Hospital, Quzhou, China

**Keywords:** paraganglioma, bladder tumor, retroperitoneal tumor, imaging, surgery

## Abstract

Paragangliomas (PGLs) are rare neuroendocrine tumors originating from chromaffin cells. The synchronous occurrence of bladder and retroperitoneal PGLs is extremely rare. This case highlights the radiological features of dual-site PGLs and their potential for misdiagnosis as bladder cancer with retroperitoneal lymph node metastasis. A 59-year-old female patient was admitted with a 2-week history of abdominal distention. Imaging showed a highly vascularized 1.3 cm × 1.2 cm nodule on the right wall of the bladder and a retroperitoneal mass measuring 1.8 cm × 3.7 cm adjacent to the left side of the abdominal aorta. Postoperative pathological examination confirmed dual-site PGLs. The multifocal appearance and hypervascular characteristics of these tumors may lead to misdiagnosis as bladder cancer with metastasis. Recognizing the clinical, imaging (such as CT and MRI findings), and pathological features is crucial for avoiding misdiagnosis and formulating an appropriate treatment plan.

## Introduction

Paragangliomas (PGLs) are neuroendocrine tumors originating from the sympathetic nerve chain outside the adrenal glands and have hormone-secreting functions. These tumors can occur at various locations, including the head, neck, chest, abdomen, and pelvis, and are also known as extra-adrenal pheochromocytomas (1). Bladder PGLs account for less than 0.1% of bladder tumors and 1% of all PGLs and often presenting as functional tumors (2, 3). Retroperitoneal PGLs account for 1% to 3% of retroperitoneal tumors and 10% of all PGLs (4). They can occur at any location in the retroperitoneum, with a predilection for the paraaortic region between the renal artery and the bifurcation of the abdominal aorta, particularly at the organ of Zuckerkandl, located between the origin of the inferior mesenteric artery and the bifurcation of the abdominal aorta (4). Bladder PGLs typically present with catecholamine-related symptoms (e.g., hypertension during urination), whereas retroperitoneal PGLs are often asymptomatic. The synchronous occurrence of tumors at these two sites is extremely rare, with only one case reported in the literature (5). Imaging plays a crucial role in diagnosis. Bladder PGLs typically appear as highly vascularized nodules with intact mucosa on CT and MRI, while retroperitoneal PGLs may show hemorrhagic necrosis and cystic changes, presenting as markedly heterogeneous enhancement. However, due to the rarity of synchronous bladder and retroperitoneal PGLs, they are frequently misdiagnosed as bladder cancer with metastasis, particularly in the absence of typical biochemical findings. Misdiagnosis as bladder cancer with metastasis can lead to inappropriate biopsy or surgery, triggering life-threatening catecholamine crises (6). Therefore, a comprehensive assessment incorporating clinical evaluation, biochemical testing, and multimodal imaging is essential.

## Case presentation

**Figure 1:**
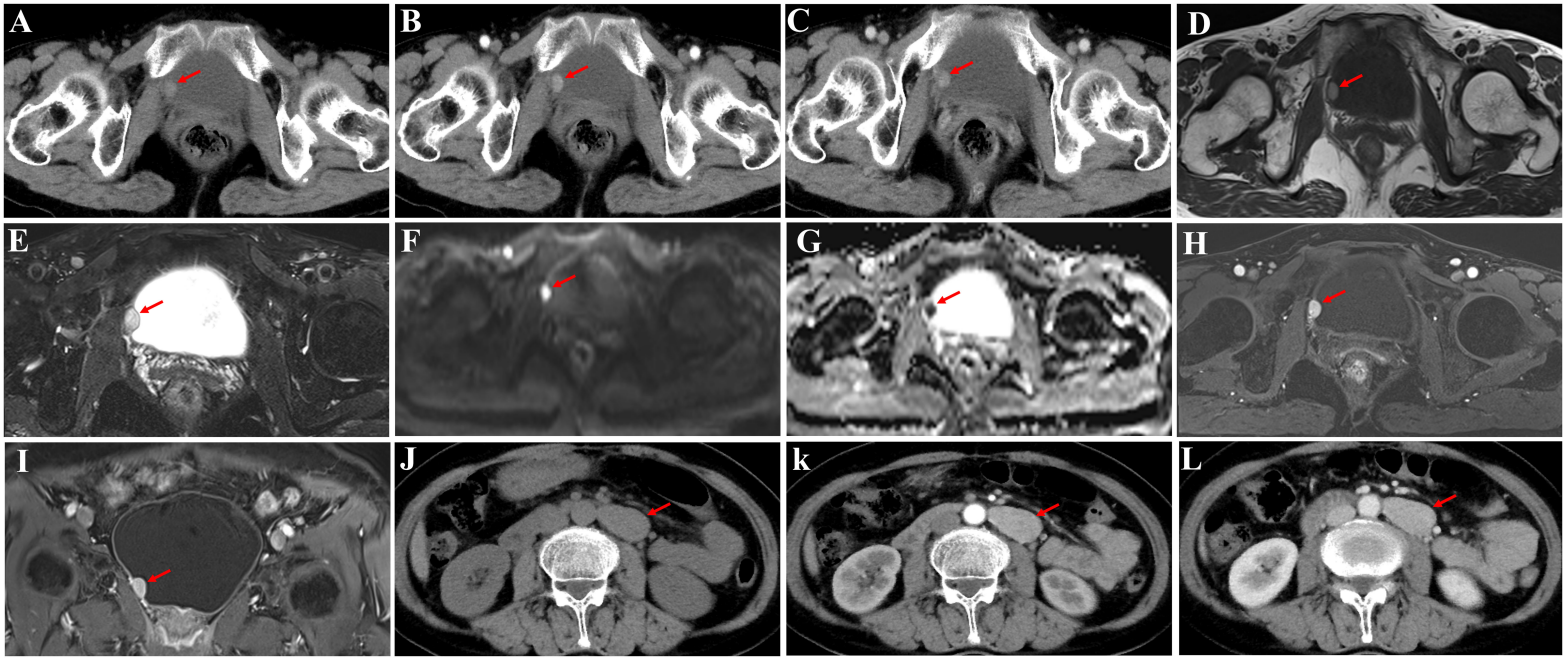
Imaging findings of a 59–year–old female patient. Bladder wall nodule **(A–I)** and left retroperitoneal tumor **(J–L)**. **(A)** A well–defined, round soft tissue nodule located on the right bladder wall, growing into the lumen, with smooth margins and homogeneous density on non–contrast CT (attenuation value: 55 HU). **(B)** Arterial–phase contrast–enhanced CT showing an attenuation value of 78 HU. **(C)** Venous–phase contrast–enhanced CT showing an attenuation value of 90 HU. **(D, E)** The lesion exhibits slightly high signal intensity on T1WI and fat–saturated T2WI, with a homogeneous signal pattern. **(F, G)** DWI shows high signal intensity, while the ADC demonstrates low signal intensity. **(H, I)** The lesion exhibits significant and persistent enhancement on contrast–enhanced imaging. **(J)** A well–defined, round soft tissue mass in the left retroperitoneum, with smooth margins and homogeneous density on non–contrast CT (attenuation value: 48 HU). **(K)** Arterial–phase contrast–enhanced CT showing an attenuation value of 97 HU. **(L)** Venous–phase contrast–enhanced CT showing an attenuation value of 110 HU. Arrow, lesion location. CT, computed tomography, T1WI, T1–weighted image, T2WI, T2–weighted image, DWI, diffusion weighted imaging, ADC, Apparent diffusion coefficient.

A 59-year-old female patient was admitted for evaluation of a 2-week history of abdominal distension. Abdominal computed tomography (CT) performed at a local hospital revealed a bladder mass of undetermined nature. Ultrasound examination showed a hypoechoic nodule on the right wall of the bladder, which was considered suspicious for bladder cancer, and cystoscopy was recommended. The patient denied visible hematuria, dysuria, frequency, urgency, painful urination, chills, fever, nausea, vomiting, or chest tightness. Her mental status was normal, and she had no history of hypertension. For further evaluation and management, the patient presented to our hospital on September 2, 2024. Upon admission, laboratory tests revealed positive hematuria (+++), while other parameters showed no significant abnormalities. Imaging studies revealed a well-defined 1.3 cm × 1.2 cm nodule on the right wall of the bladder (**Figure 1A**), demonstrating significant homogeneous and continuous enhancement on contrast-enhanced CT (**Figures 1B, C**). Magnetic resonance imaging (MRI) showed high signal intensity on T1-weighted imaging (T1WI) and fat-saturated T2-weighted imaging (T2WI) (**Figures 1D, E**), high signal intensity on diffusion-weighted imaging (DWI) (**Figure 1F**), and low signal intensity on apparent diffusion coefficient (ADC) mapping (**Figure 1G**). Arterial-phase contrast-enhanced imaging showed marked enhancement (**Figure 1H**), which slightly decreased in the venous phase (**Figure 1I**). Additionally, a 1.8 cm × 3.7 cm mass was identified adjacent to the left side of the abdominal aorta, with uniform density on unenhanced CT (**Figure 1J**) and significant homogeneous enhancement on contrast-enhanced CT (**Figures 1K, L**). The initial imaging diagnosis was bladder cancer with retroperitoneal lymph node metastasis. Subsequently, the patient underwent diagnostic transurethral resection of the bladder tumor and laparoscopic resection of the left retroperitoneal tumor. During resection of the retroperitoneal tumor, the patient experienced significant blood pressure fluctuations, with blood pressure reaching 250/120 mmHg. During resection of the bladder tumor, no significant blood pressure changes occurred. Blood pressure was carefully monitored and managed throughout the procedures, and both surgeries were successfully completed. Postoperative pathological examination confirmed the presence of synchronous PGLs in both the bladder and retroperitoneum.

**Figure 2:**
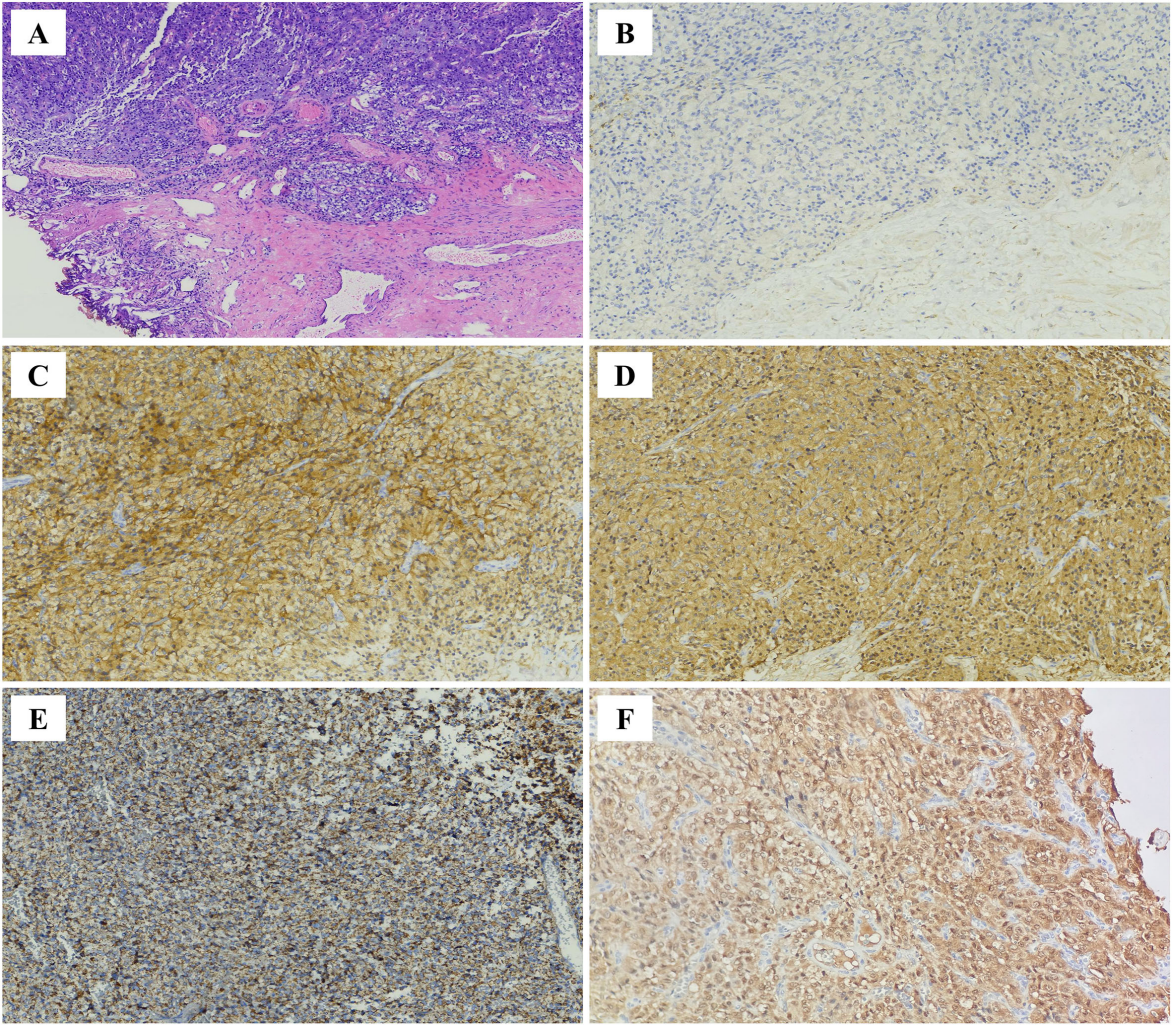
**(A)** Hematoxylin and eosin staining(HE,200×): Bladder tumor with relatively well–defined borders, small focal infiltrative growth, and focal pigment deposition. The tumor cells are arranged in short cord–like and trabecular patterns, with the stroma showing thin–walled blood sinusoids separating the cells. IHC staining of tumor cells(200×): **(B)** CK (−), **(C)** SYN (+), **(D)** CGA (+), **(E)** SDHB (+), **(F)** S100 (tumor cells +).

Microscopically, the bladder lesion demonstrates relatively well-defined tumor borders with small focal infiltrative growth pattern, and exhibits pigment deposition in focal areas. Tumor cells were round and polygonal, with granular cytoplasm. The nuclei were round to oval, with a pepper-and-salt chromatin pattern. Nucleoli were inconspicuous, and mild nuclear atypia was observed. Tumor cells were separated by fine fibrous vascular stroma and thin-walled blood sinusoids, arranged in a diffuse sheet-like pattern. Pseudo-rossette structures were observed. No necrosis was noted, and mitotic figures were absent (0-1/10 HPF). There was no evidence of neural or vascular invasion (**Figure 2A**).

**Figure 3:**
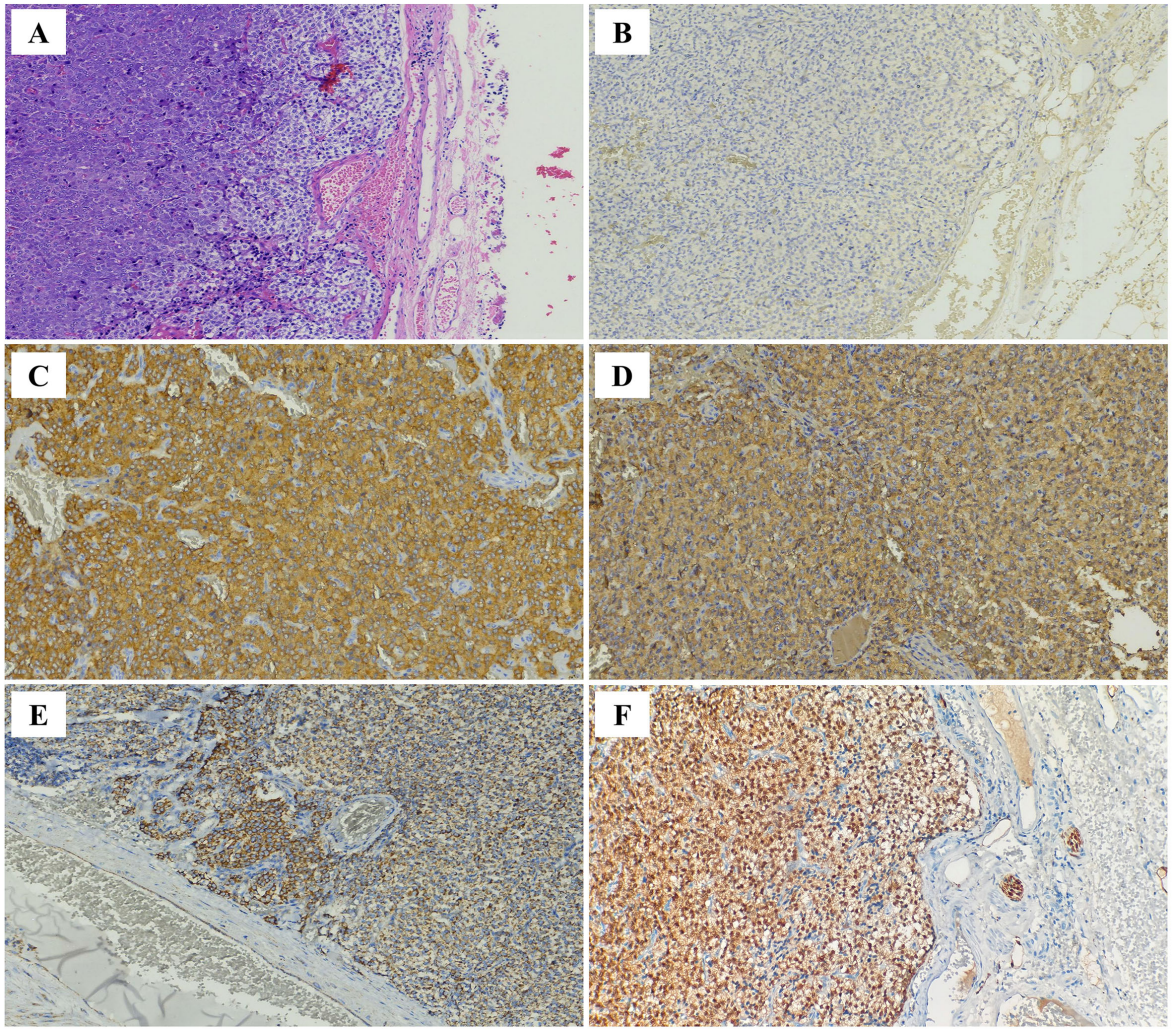
**(A)** Hematoxylin and eosin staining(HE,200×): The retroperitoneal tumor is well–capsulated with clear boundaries. Tumor cells are arranged in nest–like (Zellballen), short cord–like, trabecular, and diffuse patterns, separated by a fibrous stroma rich in blood vessels and thin–walled blood sinusoids. IHC staining of tumor cells(200×): **(B)** CK (−), **(C)** SYN (+), **(D)** CGA (+), **(E)** SDHB (+), **(F)** S100 (tumor cells +).

Microscopic examination of the retroperitoneal lesion demonstrates a well-encapsulated configuration with sharply demarcated boundaries. Tumor cells were also round and polygonal, with granular cytoplasm. The nuclei were round or oval, exhibiting a pepper-and-salt chromatin pattern, and nucleoli were inconspicuous, with mild to moderate nuclear atypia. The tumor cells were surrounded by a peripheral rich network of thin-walled blood sinusoids, with some areas showing a densely packed nest-like structure. Tumor cells were arranged in short trabecular, small cord-like, and diffuse sheet-like patterns, with pseudo-rossette structures observed. No necrosis was noted, and mitotic figures were absent (0-1/10 HPF). There was no evidence of neural or vascular invasion (**Figure 3A**).

Immunohistochemical (IHC) staining (**Figures 2**, **3**) for both lesions showed positivity for CD56, synaptophysin (Syn), chromogranin A (CgA), Vimentin, SDHB, S100(tumor cells), and fumarate hydratase (FH), and negativity for cytokeratin (CK), SOX10, GATA3, carbonic anhydrase IX (CAIX), and α-inhibin. The Ki-67 proliferation index was low (<1%, <20/MPF). Based on histological and IHC findings, the tumors in both the bladder and retroperitoneum were classified as paragangliomas (PGLs). The GAPP (grading system for adrenal pheochromocytoma and paraganglioma) score for both lesions was 3, indicating moderate differentiation. The patient had an uneventful postoperative recovery. Further questioning revealed no family history of PGLs. Follow-up to date shows normal laboratory results, including catecholamine levels and urinalysis, with no signs of recurrence or metastasis on imaging studies.

## Discussion

PGLs are neuroendocrine tumors originating from the sympathetic nerve chain outside the adrenal glands and possess hormone-secreting potential. Functional PGLs synthesize, secrete, and release large amounts of catecholamines (e.g., norepinephrine, epinephrine, dopamine), leading to hypertension, metabolic alterations, and a spectrum of clinical syndromes. These include cardiovascular, cerebrovascular, renal, and vascular complications, which can be life-threatening. PGLs are commonly located along the paravertebral sympathetic chain in the thoracic, abdominal, and pelvic regions (7–9). PGLs are classified as functional or non–functional (10). Functional PGLs secrete catecholamines, leading to symptoms such as hypertension, palpitations, and headaches (11). Non–functional PGLs typically cause symptoms only due to mass effect from significant growth compressing adjacent structures or are discovered incidentally during imaging studies (12). In this case, the patient did not exhibit significant hypertension or metabolic changes prior to surgery, and there was no notable blood pressure fluctuation during the resection of the bladder lesion. However, during the retroperitoneal tumor resection, the patient experienced significant blood pressure fluctuations, with the highest value reaching 250/120 mmHg. This may have been due to surgical manipulation of the tumor or surrounding tissues, which could have stimulated the adjacent sympathetic nerves, leading to sympathetic nervous system activation.

Differentiating functional from non–functional PGLs primarily relies on biochemical testing and functional imaging studies. Functional PGLs are characterized by elevated levels of catecholamines and/or their metabolites in urine or plasma, detectable through measurements of urinary fractionated metanephrines, plasma–free metanephrines, or functional imaging such as ^123^I–metaiodobenzylguanidine (MIBG) scintigraphy (13, 14). Non–functional PGLs typically do not show elevated catecholamine levels and are often found incidentally during other clinical investigations (3). In this case of extremely rare synchronous bladder and retroperitoneal PGLs, the patient lacked significant clinical abnormalities suggestive of functionality. Consequently, the possibility of PGLs was not considered preoperatively, and relevant biochemical or functional imaging studies were not performed. Additionally, the patient lacked typical symptoms of functional PGLs (e.g., paroxysmal hypertension, headaches, palpitations, sweating, syncope) preoperatively. Therefore, the final diagnosis favored non–functional PGLs. Imaging plays a critical role in the diagnosis of PGLs. Bladder PGLs typically appear as highly vascularized nodules on CT and MRI, showing significant arterial–phase enhancement and persistent or progressive delayed enhancement, features that can overlap with bladder cancer. On MRI, diffusion–weighted imaging (DWI) and apparent diffusion coefficient (ADC) maps typically show restricted diffusion, resembling bladder cancer. However, bladder PGLs typically arise within the bladder wall (submucosa/muscularis propria), preserving an intact mucosal surface. On dynamic contrast–enhanced MRI, early arterial phase images may show a uniform high signal intensity mass beneath an intact mucosal lining, differentiating it from the nodular or cauliflower–like masses typical of bladder cancer that often involve the mucosa (15–17). Retroperitoneal PGLs are prone to hemorrhage, necrosis, and cystic changes, leading to heterogeneous enhancement. These tumors may also compress nearby organs, such as the kidneys and major blood vessels. Large tumors adjacent to the pancreas may be misdiagnosed as pancreatic malignancies (18, 19). In this case, the retroperitoneal mass had uniform density on unenhanced CT, without obvious hemorrhage, necrosis, or cystic changes. Contrast–enhanced scans revealed significant, homogeneous enhancement, which is an atypical imaging feature for retroperitoneal PGLs, making the diagnosis challenging. Given the high prevalence of bladder cancer, the patient was initially misdiagnosed with bladder cancer with retroperitoneal lymph node metastasis.

The synchronous occurrence of bladder and retroperitoneal PGLs is extremely rare, with only one case reported in the literature (5). In that report, both the bladder and retroperitoneal masses were non–functional PGLs. The CT findings of the bladder wall lesion were similar to those in the present case, with significant homogeneous enhancement on contrast–enhanced imaging. However, the case report did not include MRI data, preventing a comparison of MRI images between the two cases. In this case, pelvic MRI showed high signal intensity on T1WI and fat–saturated T2WI, as well as restricted diffusion on DWI and low ADC values, consistent with the solid, hypervascular nature of the tumor. Pathological examination confirmed that the tumors exhibited a solid sheet–like pattern, with abundant vascular and blood sinus structures. The retroperitoneal mass in the literature case was larger (6.5 cm × 5.4 cm), with heterogeneous enhancement and central necrosis, which is characteristic of retroperitoneal PGLs. However, in this case, the retroperitoneal mass was smaller (1.8 cm × 3.7 cm), with uniform density and no significant signs of necrosis, making it more likely to be misdiagnosed as retroperitoneal lymphadenopathy, which warrants clinical vigilance.

Histopathologically, PGLs are typically characterized by chief cells and sustentacular cells, embedded within a rich network of thin–walled capillaries, forming the characteristic organoid nesting pattern known as “zellballen”. Tumor cells (chief cells) are often arranged in patterns such as nests (zellballen), trabeculae, cords, or solid sheets. Pseudorosette formation may also be present. The tumor cells are predominantly uniform in shape, being round or polygonal, with granular cytoplasm that may be eosinophilic, basophilic, or amphophilic. The nuclei are round, oval, or vesicular with a pepper–and–salt chromatin pattern. Necrosis and mitotic figures are rare. The stroma contains fibrovascular tissue and thin–walled sinusoidal vessels (20). In this case, although both lesions predominantly exhibited a solid sheet–like growth pattern of tumor cells, typical organoid (zellballen) structures were not prominent. However, the cellular morphology and characteristics were consistent with PGLs. IHC analysis plays an essential role in the diagnosis and classification of PGLs. Neuroendocrine neoplasms are broadly categorized into epithelial neuroendocrine tumors (NETs) and neural/paraganglionic tumors, with PGLs belonging to the latter group. PGLs are typically positive for neuroendocrine markers such as CD56, synaptophysin (Syn), chromogranin A (CgA), and insulinoma–associated protein 1 (INSM1), and negative for cytokeratins (CK). Epithelial neuroendocrine tumors (NETs) typically exhibit diffuse cytokeratin (CK) positivity (21). GATA3 (GATA–binding protein 3), a member of the GATA zinc finger transcription factor family, shows a high expression rate (approximately 80%) in PGLs (22). However, its specificity in differential diagnosis is relatively limited, as it is widely expressed in other tumor types, including breast cancer and urothelial carcinoma, and thus cannot be used to distinguish bladder PGLs from urothelial carcinoma. Literature reports indicate that GATA3 expression in PGLs can be variable, showing weak positivity or negativity in some cases, potentially due to methodological differences in staining and interpretation (23). In the current case, GATA3 was negative in both lesions. However, CD56, Syn, and CgA were diffusely positive, and CK was negative. Considering the lesion locations and the histopathological features, the diagnosis of PGLs at both sites was confirmed.

Beyond differential diagnosis, IHC is also crucial for prognosis evaluation and risk stratification of PGLs. S100 and SOX10 stain the sustentacular cells in PGLs and are usually positive in the majority of functional PPGLs (21, 24). These markers have historically been used for differentiating PGLs. However, S100/SOX10 positivity can occasionally occur in tumor cells themselves, and other neuroendocrine tumors (e.g., some NETs) may also harbor sustentacular cells. Therefore, S100 and SOX10 are no longer considered essential IHC markers for diagnosing PGLs, though they are still useful for prognosis evaluation, as their expression is significantly reduced in more invasive PGLs. SDHB immunohistochemistry is an important surrogate marker for detecting SDHx–related pathogenesis. Loss of SDHB expression is indicative of SDHx gene–associated tumors (25). A subset of PGLs harbor SDHB gene mutations, and carriers are at increased risk for hereditary tumor syndromes, recurrence, and malignancy. SDHB gene mutations are considered high–risk factors for malignancy and recurrence in PGLs (26). Fumarate hydratase (FH) deficiency leads to fumarate accumulation, impaired mitochondrial oxidative phosphorylation, enhanced glycolysis (supporting tumor growth), and impaired DNA repair (27). Studies have shown that FH–deficient PGLs have higher malignancy and metastasis rates. Patients with von Hippel–Lindau (VHL) syndrome have an approximately 20% lifetime risk of developing PGLs. SDHx gene mutations are the most common cause of hereditary PGLs, followed by pathogenic VHL gene variants (26, 27). Carbonic anhydrase IX (CAIX) is a highly specific IHC marker for VHL–associated PGLs, while α–inhibin is a sensitive marker for PGLs associated with SDHx or VHL mutations (28).The Ki–67 proliferation index, a commonly used marker for evaluating cellular proliferation activity, is significantly correlated with prognosis in pheochromocytoma and PGLs patients (26). Previous studies have indicated that a Ki–67 index ≥3% in PPGLs is associated with increased risks of postoperative recurrence and metastasis (29). In the present case, both lesions showed retained S100(tumor cells),SDHB and FH expression, were negative for SOX10, CAIX, and α–inhibin, and had a Ki–67 index of <1%. These findings suggest a favorable prognosis and place the patient in a low–risk stratification group. In addition to IHC, risk assessment for PGLs can also be aided by the use of the adrenal pheochromocytoma and paraganglioma grading system (GAPP) proposed by Kimura et al., which is widely used for risk stratification of PGLs. The system is based on histological criteria, including morphological features, cellular structure, coagulative necrosis, vascular or capsular invasion, and is combined with the Ki–67 proliferation index and catecholamine secretion type to provide an integrated scoring system. In this system, GAPP scores of 0–2 correspond to well–differentiated tumors, 3–6 to moderately differentiated tumors, and 7–10 to poorly differentiated tumors (24, 30). The GAPP score for both lesions in this case was 3, indicating moderately differentiated tumors.

The World Health Organization (WHO) classification system groups paragangliomas (PGLs) and pheochromocytomas (PCCs) together as pheochromocytomas/paragangliomas (PPGLs). Prior to the 2017 WHO classification of endocrine tumors, PPGLs were often designated as “benign” or “malignant”. However, the 2017 WHO classification considers all PPGLs to have metastatic potential and recommends categorizing them based on the presence or absence of metastases (i.e., metastatic or non–metastatic) (31). This classification was maintained in the 2022 WHO update (21). Clinical studies indicate that metastatic PPGLs account for approximately 10–17% of all cases, with PGLs constituting 15–35% of metastatic PPGLs (32).For multifocal PGLs, the primary challenge is distinguishing between primary PGLs with metastasis and synchronous PGLs. The differential diagnosis primarily relies on imaging and pathological examination. In this case, the bladder and retroperitoneal lesions were considered synchronous PGLs, which needed to be distinguished from primary bladder PGLs with retroperitoneal metastasis. From an imaging perspective, both lesions were small, well–defined, and exhibited homogeneous attenuation/signal intensity. The retroperitoneal lesion had a complete capsule, and there were no signs of hemorrhage, necrosis, cystic changes, or calcification. Additionally, there was no evidence of invasion into adjacent organs or tissues, or lymph node enlargement, suggesting a low–grade malignant tumor. Preoperative imaging, including cranial, chest, abdominal, and pelvic CT scans, as well as neck ultrasound, revealed no other space–occupying lesions aside from the bladder and retroperitoneal masses. Therefore, imaging findings did not support a diagnosis of bladder paraganglioma with retroperitoneal metastasis. Pathological examination revealed no evidence of necrosis or neurovascular invasion in either lesion. Immunohistochemically, both lesions demonstrated retained expression of SDHB and FH, with S100 positivity in tumor cells, while exhibiting negativity for SOX10, GATA3, CAIX, and α–inhibin. The Ki–67 proliferation index was less than 1% in both specimens. A GAPP score of 3 was assigned to each lesion, indicating low malignant potential and low metastatic risk. According to the WHO classification, metastatic PPGLs are defined by the presence of tumors in non–chromaffin (non–paraganglionic) sites, such as bone, liver, lung, lymph nodes distant from the primary, brain, or soft tissue (29). As both lesions in this case are located within known paraganglionic sites (bladder and retroperitoneum), they do not meet the criteria for metastatic disease. Considering all these factors, the final diagnosis was synchronous primary PGLs of the bladder and retroperitoneum, rather than metastatic disease. This diagnosis is supported by the imaging and pathological findings, which demonstrate characteristics consistent with primary PGLs, with no evidence of metastatic spread.

In conclusion, this case highlights the importance of recognizing the imaging and pathological features of synchronous PGLs to avoid misdiagnosis and ensure appropriate treatment planning. Bladder and retroperitoneal PGLs should be considered in the differential diagnosis of multifocal, highly vascular tumors, and diagnosis should be confirmed through histopathological and IHC examination to prevent misdiagnosis as bladder cancer with retroperitoneal metastasis or bladder PGLs with retroperitoneal metastasis. Accurate diagnosis and timely surgical intervention are crucial to avoid the risks associated with misdiagnosis and ensure optimal patient outcomes.

## Data Availability

The original contributions presented in the study are included in the article/supplementary material. Further inquiries can be directed to the corresponding author.
